# High prevalence of malnutrition among patients with solid non-hematological tumors as found by using skinfold and circumference measurements

**DOI:** 10.1590/S1516-31802005000600005

**Published:** 2005-11-01

**Authors:** Adriana Garófolo, Fábio Ancona Lopez, Antonio Sérgio Petrilli

**Keywords:** Malnutrition, Nutritional assessment, Anthropometry, Cancer, Child, Adolescent, Desnutrição, Avaliação nutricional, Antropometria, Câncer, Criança, Adolescente

## Abstract

**CONTEXT AND OBJECTIVE:**

Malnutrition in cancer patients has many causes. Nutritional status is usually assessed from weight/height indices. These present limitations for the nutritional assessment of cancer patients: their weights include tumor mass, and lean mass changes are not reflected in weight/height indices. The objective was to evaluate differences between two anthropometric methods and compare deficits, in non-hematological tumor patients and hematological disease patients.

**DESIGN AND SETTING:**

Cross-sectional study at Instituto de Oncologia Pediátrica, Universidade Federal de São Paulo.

**METHODS:**

Children and adolescents were evaluated between March 1998 and January 2000. Traditional anthropometric measurements were obtained in the first month of treatment (induction therapy), by weight-for-height (W/H) using z-scores index for children and body mass index (BMI) for adolescents. Body composition evaluations consisted of specific anthropometric measurements: triceps skinfold thickness (TSFT), mid-upper arm circumference (MUAC) and arm muscle circumference (AMC). Data were analyzed to compare nutritional assessment methods for diagnosing malnutrition prevalence. The chi-squared test was used for comparative analyses between tumor patients and hematological disease patients.

**RESULTS:**

Analysis was done on 127 patients with complete data. Higher percentages of deficits were found among tumor patients, by W/H z-scores or BMI and by MUAC and AMC. Higher percentages of deficits were shown by TSFT (40.2%) and MUAC (35.4%) than by W/H z-scores or BMI (18.9%).

**CONCLUSION:**

Non-hematological tumor patients presented higher malnutrition prevalence than did hematological disease patients. Body composition measurements by TSFT and MUAC detected more patients with malnutrition than did W/H or BMI.

## RESUMO

**CONTEXTO E OBJETIVO:**

Em pacientes com câncer, a desnutrição tem múltiplas causas. Medidas antropométricas de peso e estatura são o método mais utilizado para avaliação do estado nutricional. Infelizmente, esse método é limitado em pacientes com câncer, pois o peso inclui o tumor e a relação peso/estatura não leva em conta alterações específicas de tecido magro. O objetivo deste estudo é avaliar diferenças entre medidas antropométricas e de composição corporal em crianças e adolescentes com câncer.

**TIPO DE ESTUDO E LOCAL:**

Estudo de corte transversal realizado no Instituto de Oncologia Pediátrica, Universidade Federal de São Paulo — Escola Paulista de Medicina, São Paulo, Brasil.

**MÉTODOS:**

Crianças e adolescentes com câncer com idade acima de um ano foram avaliadas de março de 1998 a janeiro de 2000. Medidas antropométricas tradicionais foram coletadas no primeiro mês de tratamento oncológico (terapia de indução) por meio do escore-z de peso para estatura (P/E) nas crianças e índice de massa corpórea (IMC) nos adolescentes. A avaliação da composição corporal foi composta por medidas de prega cutânea triciptal (PCT), circunferência do braço (CB) e circunferência muscular do braço (CMB). Os dados foram analisados comparando-se as prevalências de desnutrição entre os métodos de avaliação nutricional. O teste do qui-quadrado e o grau de associação foram usados para comparar as taxas entre portadores de tumores sólidos e hematológicos.

**RESULTADOS:**

139 pacientes foram avaliados, 127 tinham dados completos para análise. O estudo demonstrou maior percentual de déficit nos portadores de doenças sólidas não-hematológicas pelo P/E ou IMC, CB, e CMB. A análise global também sugere que a PCT (40%) e a CB (35%) demonstraram maior percentual de déficit quando comparadas ao escore-z de P/E ou IMC (19%).

**CONCLUSÃO:**

Pacientes com tumores sólidos apresentaram maior prevalência de desnutrição. As medidas de composição corporal por meio da PCT e CB detectaram mais pacientes desnutridos do que o P/E e o IMC.

## INTRODUCTION

Malnutrition in cancer patients is related to factors associated with the treatment and with the disease itself, and others such as the economic and social conditions. Food intake, energy expenditure and nutrient absorption and metabolism, as well as complications such as oral and gastrointestinal toxicity and nephrotoxicity caused by drugs used to treat neoplasias and infections play an important role in the etiology of malnutrition in cancer.^1^

Food intake and appetite alterations have been identified as some of the main causes of malnutrition. The acceptance of foods is influenced by emotional and psychological factors, in addition to those associated with the treatment and the disease itself.^2^ Metabolic disturbance is another problem among cancer patients, and this is often represented by catabolic status. It has been shown that the weight losses that occur in cachexia lead to reductions in lean body tissue.^3,4^

Among children and adolescents, the treatment itself, and particularly chemotherapy and radiotherapy, seems to be an important nutritional risk factor. Their treatment is associated with nausea and vomiting, oral mucositis, constipation, xerostomia, dysgeusia and food aversion, and it thus plays an important role in decreased food intake, nutrient loss, energy expenditure alterations and weight loss, particularly lean body mass.^5^ These conditions predispose such patients towards malnutrition, especially when there are frequent periods of chemotherapy treatment.^6^

Because of weight variations that are associated with tumor size, other methods should be utilized to identify malnutrition, in addition to the weight-for-age and weight-for-height methods. Thus, the present study was carried out with the objective of evaluating and comparing two simple anthropo-metrical methods for assessing the nutritional status in children and adolescents with cancer, with comparison of the deficits between solid non-hematological and hematological malignancy diseases at the beginning of the induction therapy.

## PATIENTS AND METHODS

The subjects were children aged over one year and adolescents who were evaluated between March 1998 and January 2000 within the support group for children with cancer [Grupo de Apoio à Criança com Câncer (GRAACC)] at the Pediatric Oncology Institute [Instituto de Oncologia Pediátrica (IOP)], Universidade Federal de São Paulo. The patients were evaluated during their first month of treatment, in the form of a cross-sectional study while they were undergoing therapy to induce clinical remission. They were divided according to their disease type (solid non-hematological tumors and hematological malignancies).

The inclusion criteria for subjects were that they should be children and adolescents referred to Instituto de Oncologia Pediátrica with a diagnosis of malignant disease; they should be aged over one year; and a dietitian should have made an initial assessment.

The exclusion criteria for subjects were that they should not present any cancer-related diseases such as diabetes mellitus, cardiopathy, chronic obstructive pulmonary disease (COPD), gastrointestinal diseases, nephropathy etc; and they should not be relapsed patients.

### Nutritional assessment

The weight-for-height (W/H) z-score was classified in accordance with the World Health Organization (WHO) 1999 criteria for malnutrition in children,^7^ and the body mass index (BMI) percentiles in accordance with the WHO 1995 criteria for malnutrition in adolescents.^8^

Triceps skinfold thickness (TSFT), mid-upper arm circumference (MUAC) and arm muscle circumference (AMC) were measured at the same time, during the first month of treatment, in the first chemotherapy cycle. TSFT was determined by grasping the skin and adjacent subcutaneous tissue between the thumb and forefinger, shaking it gently to exclude underlying muscle, and pulling it away from the body just far enough to allow the jaws of the caliper (Harpenden and Cescorf models) to impinge on the skin. Duplicate readings were made at this site to improve the accuracy and reproducibility of the measurements. MUAC was determined at the midpoint between the acromion and olecranon. From these two measurements, AMC was calculated as follows:


AMC=MUAC−(TSFT x 0.314)

These variables were interpreted in accordance with the Frisancho (1993) percentiles charts^9^ and their percentages of adequacy were demonstrated, which were obtained as follows:


$$\text{Percentage adequacy}=\frac{\text{Observed value}}{\text{Ideal value}}\text{X}100$$

The oncological treatment protocols used consisted of chemotherapy, radiotherapy and surgery, depending on the tumor diagnosis (Table 1).

**Table 1.** Diagnoses of 127 children and adolescents with cancer (72 boys and 55 girls), studied in Sao Paulo

Diagnosis	Proposed treatment protocol	n
**Solid non-hematological tumors**		**68**
Osteosarcoma	GBCTTO: doxorubicin, carboplatin, cisplatin, ifosfamide and surgery	26
Central nervous system tumors		
Astrocytomas	carboplatin, vincristine and surgery	7
Medulloblastomas	ifosfamide, etoposide, irradiation and surgery	3
Craniopharyngiomas	bleomycin and surgery	2
Choroid plexus carcinoma	ifosfamide and surgery	1
Rhabdoid cerebral sarcoma	surgery	1
Neuroblastomas III and IV (high risk)	CAP-ICE: cyclophosphamide, doxorubicin, platinum and ifosfamide, carboplatin, etoposide and surgery	9
Wilms’ tumor III and IV	actinomycin D, vincristine, adriblastin, surgery and/or radiation	6
Rhabdomyosarcoma	IRS: Surgery, radiation, vincristine, doxorubicin/actinomycin, cyclophosphamide, ifosfamide and vepesid	3
Ewing's sarcoma	IESS-II: vincristine, actinomycin D, cyclophosphamide, doxorubicin, ifosfamide and etoposide; surgery and local radiation	2
Others		
Primitive neuroectodermal tumor		2
Liver sarcoma		2
Germ cell tumor		1
Leiomyosarcoma		1
Renal carcinoma		1
Adenocarcinoma		1
**Hematological malignancies**		**59**
Acute lymphocytic leukemia	GBTLI LLA-93. Induction therapy for basic risk patients: dexamethasone, vincristine, daunorubicin and MADIT (methotrexate, cytarabine and intrathecal dexamethasone). For high risk patients: dexamethasone, vincristine, daunorubicin, L-asparaginase and MADIT. Intensification therapy: 6-mercaptan, methotrexate and MADIT.	32
Acute myelocytic leukemia	LMAIO-97. Induction therapy: 2-CDA, cytarabine (experimental therapy), MADIT, DAV-1 and DAV-2 (dau- norubicin, cytarabine and etoposide). Consolidation therapy: cytarabine, mitoxantrone and etoposide.	8
Non-Hodgkin's lymphoma	Low risk: prednisone, cyclophosphamide, vincristine and methotrexate. High risk: B-cell lymphoma: prednisone, cyclophosphamide, vincristine, methotrexate, VM 26, cytarabine, etoposide, ifosfamide and adri- blastin. T-cell lymphoma: prednisone, dexamethasone, intrathecal methotrexate, vincristine, daunorubicin, l-asparaginase, 6-mercaptan, cyclophosphamide and cytarabine.	12
Hodgkin's disease	MOPP (mechlorethamine, vincristine, procarbazine and prednisone) or ABVD (doxorubicin, bleomycin, vinblastine and dacarbazine) and local radiation.	7
**Total**		**127**

GBCTTO: Brazilian Cooperative Group for Bone Tumor Treatment; IRS: Intergroup Rhabdomyosarcoma Study; IESS-II: Intergroup Ewing's Sarcoma Study II; GBTLI LLA-93: Brazilian Treatment Group for Childhood Leukemia; LMAIO-97: International Outreach.

The Medical Ethics Committee of Universidade Federal de São Paulo gave its approval for the nutritional study protocol. The corresponding consent from all the subjects’ parents or guardians was obtained after the study protocol had been explained to them.

### Data analysis

Data were analyzed qualitatively by percentage values, to compare the nutritional assessment methods for diagnosing malnutrition. The Pearson chi-squared test and its degree of association by the phi (φ) coefficient was utilized for comparative analyses between the two groups (solid tumors and hematological disease) and between the deficits found using z-scores and other methods. A p-value of less than 0.05 was considered significant.^10^

## RESULTS

Out of a total of 137 patients evaluated, 127 presented complete data and were used in the analysis. The evaluation was done during the first month of treatment, in the first chemotherapy cycle of the induction therapy. Among the 127 patients analyzed, 68 (53.5%) had solid tumors and 59 (46.5%) hematological malignancies; 56.7% were male and 43.3% female. The mean and median ages were 8.98 ± 5.77 and 6.67 years (range: 1.92 – 24.58 years) for the hematological disease group and 9.72 ± 6.42 and 10.5 years (range: 1.08 – 23.75 years) for the solid tumor group, respectively. Table 1 shows the cancer diagnoses of the 127 patients.

The comparative analysis of deficits according to z-scores and TSFT, MUAC and AMC demonstrated significant differences between z-score and TSFT, and z-score and MUAC (p < 0.05). Thus, the overall analysis showed significantly higher percentages of deficits using TSFT (40.2%) and MUAC (35.4%) than when using W/H z-scores or BMI (18.9%). Patients with solid tumors presented greater deficits than did those with hematological malignancy diseases, but statistical differences were observed only for z-score, TSFT and AMC, with a low degree of association (Table 2). It was further observed that TSFT, MUAC and AMC identified greater numbers of malnourished patients than did the z-score, particularly in the hematological disease group.

**Table 2.** Distribution of 127 malnourished children and adolescents according to tumor group and nutritional assessment methods

Hematological malignancies		n = 59	Solid non-hematological tumor		n = 68	Statistical analyses*	
Malnutrition criterion	Number	Percentage	Malnutrition criterion	Number	Percentage	Chi-squared	Phi coefficient
Z-score/BMI	4	6.8	z-score/BMI	20	29.4	p < 0.05	0.28
TSFT	20	33.9	TSFT	31	45.6	NS	0.11
MUAC	15	25.4	MUAC	30	44.1	p < 0.05	0.18
AMC	6	10.2	AMC	23	33.8	p < 0.05	0.28

*Statistical differences between solid non-hematological tumors and hematological malignancies; BMI = body mass index; TSFT = triceps skinfold thickness; MUAC = mid-upper arm circumference; AMC = arm muscle circumference; Comparative analysis of z-score deficits with TSFT, MUAC and AMC: z-score versus TSFT: p < 0.05; z-score versus MUAC: p <0.05; z-score versus AMC: p = NS (not significant).*

## DISCUSSION

Many studies have demonstrated that patients with cancer are malnourished. Sanchez et al. (1992) found a malnutrition rate of 14% from weight-for-height indices and 47% from biochemical indices. However, patients with solid tumors seem to be more prone to developing malnutrition.^11^ A study by Schiavetti et al. (2001) found a malnutrition rate of 26% from weight-for-height indices among Italian children with solid tumors, during antineo-plastic therapy.^12^ Elhasid et al. (1999) evaluated biochemical indices rather than anthropometric indices among 50 children with solid tumors.^13^ They observed that 36% of them had prealbumin levels that were lower than normal at the time of diagnosis. Another study, among osteosarcoma patients only, demonstrated that malnutrition prevalence increased during oncological treatment, as measured both by BMI and by TSFT, MUAC and AMC.^14^

Our results have demonstrated that the z-score or BMI and the AMC and MUAC indexes were, statistically, significantly more depleted among children and adolescents with solid tumors than with hematological diseases. Patients with solid tumors seem to be nutritionally depleted, while those with lymphoma and leukemia tend to demonstrate fewer nutritional problems. This effect may be associated with the nature of the disease as well as with the therapy.

Hematological malignancies consist of cells that proliferate rapidly, and this characteristic enables earlier diagnosis and greater response to chemotherapy.^15^ On the other hand, in the present study, the children and adolescents with hematological disease received corticosteroids as therapy to induce remission. These drugs are associated with several side effects: the effects most relevant to nutritional status are increased appetite, gains in fat mass and loss of muscle protein.^16^ In spite of this, in the present study we did not observe statistical differences in TSFT between patients with solid tumors and hematological malignancies, but the percentage deficit was higher for the solid tumor group. Because corticosteroids improve fat stores further, in order to increase the catabolism of muscle protein, it should be expected that more differences would be found in fat tissue.^16^ On the other hand, catabolism of lean body mass is associated with a poor quality of life, co-morbidity factors like infection and organ complications, and increased mortality risk.^17^ Children and adolescents with cancer, especially solid tumors, have reduced body protein stores, due to greater whole-body protein breakdown. This catabolic state may occur as a consequence of the malignant disease itself, anticancer therapy or complications of the therapy, e.g. infections and organ failure.^18^ Therefore, catabolism of lean body mass is a common effect of the disease, thereby making the evaluation of body composition an essential part of the assessment of such patients. Furthermore, children and adolescents with cancer present problems with their weight measurements because of tumor size, amputation and sometimes edema. Thus, measurement of body composition is an important procedure in evaluating patients in several situations, especially with regard to catabolic diseases.^19^

Although there are numerous methods for such assessments, the majority are too expensive and impractical for day-to-day use at the bedside or in the field. However, the anthropometric parameters of TSFT and circumferences are practical and, if properly measured, may provide good nutritional indicators in pediatric cancer populations.^20^

Smith et al. (1991) observed a malnutrition prevalence of 5% at the time of diagnosis, among children and adolescents with cancer, as measured by the z-scores of weight-for-height and height-for-age. However, in the same study, 20 and 23% of the patients were depleted, accordance to MUAC and TSFT, respectively. These results thus demonstrated that anthropometry of the arm was more efficacious for detecting early malnutrition.^21^

In the present study, we observed that weight measurements underestimated the malnutrition prevalence among children and adolescents with cancer, in comparison with TSFT, MUAC and AMC. Brennan et al.^22^ compared three methods for evaluating nutritional status. They demonstrated that weight and height overestimated the status, while anthropometry of the arm was shown to be independent of tumor size and, hence, was a better indicator of nutritional status. Their findings indicated positive correlation between MUAC or skin-fold thickness and insulin-like growth factor I (IGF-I). In the same study, they observed that solid tumor patients were more depleted, as measured by MUAC and skinfold thickness, than were leukemia patients. The prevalence analysis showed that 1 out of the 12 children with acute leukemia was malnourished, but 13 out of the 26 children with solid tumors fulfilled the definition of malnutrition.

Our results corroborate those of Brennan et al. (1999),^22^ and have also demonstrated that the AMC and MUAC indices were, statistically, significantly more depleted among children and adolescents with solid tumors than among children and adolescents with hematological diseases.

Taskinen & Saarinen-Pihkala (1998)^23^ evaluated the nutritional status of children with solid tumors, at the time of diagnosis and during preoperative and postoperative chemotherapy. They found that the traditional methods (weight-for-height, loss of weight and albumin) for assessing protein energy malnutrition did not detect more than two-thirds of the patients with reduced muscle protein mass, as indicated by muscle thickness ultrasonography. However, midarm muscle area and prealbumin had the best validity.

Thus, our study has confirmed the results of Brennan and others regarding nutritional status in solid tumor cases, and has shown the importance of body composition measurements in both the solid tumor and hematological disease groups. Our study has also demonstrated that the fat tissue and circumference indices were more powerful indicators of nutritional deficits than were weight indices, especially when using the WHO definitions for the classifications. This may be associated with the corticosteroid therapy that the hematological group underwent and the weight of the tumor in the other cancer patients. Nonetheless, additional factors such as the sensitivity and specificity of the methods should also be investigated.

## CONCLUSIONS

Patients with solid tumors presented higher malnutrition prevalence than those with hematological tumors. It is imperative that clinicians and dieticians be aware of the need for more accurate assessment of nutritional status in children and because of the catabolism of the disease, and the side effects of the oncological treatment, particularly in muscle tissue. TSFT and MUAC detected a greater number of cancer patients with malnutrition than did W/H or BMI.
